# Draft Genome Sequence of Pseudomonas sp. Strain B1, Isolated from a Contaminated Sediment

**DOI:** 10.1128/genomeA.00518-18

**Published:** 2018-06-21

**Authors:** Ashish Pathak, Rajneesh Jaswal, Paul Stothard, Scott Brooks, Ashvini Chauhan

**Affiliations:** a School of the Environment, Florida A&M University, Tallahassee, Florida, USA; b Department of Agricultural, Food and Nutritional Science, University of Alberta, Edmonton, AB, Canada; c Environmental Sciences Division, Oak Ridge National Laboratory, Oak Ridge, Tennessee, USA

## Abstract

The draft genome sequence of Pseudomonas sp. strain B1, isolated from a contaminated soil, is reported. The genome comprises 6,706,934 bases, 6,059 coding sequences, and 70 RNAs and has a G+C content of 60.3%. A suite of biodegradative genes, many located on genomic islands, were identified from strain B1, further enhancing our understanding of the versatile pseudomonads.

## GENOME ANNOUNCEMENT

Large amounts of mercury (Hg) were used at the U.S. Department of Energy’s Y-12 facility in Oak Ridge, TN, from the 1950s to the early 1960s (1). Consequently, East Fork Poplar Creek (EFPC), which flows through the city of Oak Ridge, and its floodplain were left contaminated with Hg (2). To assess genome-wide biodegradative traits in microorganisms native to EFPC, sediment cores were collected in December 2017 from a location approximately 5 kilometers upstream of the creek mouth. Cores were shipped on ice to the Florida A&M laboratory, where sediments were serially diluted and plated onto Luria-Bertani (LB) medium containing Hg (5 µg/ml, provided as mercuric chloride) to isolate Hg-resistant bacteria. One robustly growing strain, tentatively labeled as B1, was chosen for genome sequencing.

Genomic DNA from strain B1 was extracted, processed according to the Nextera XT library preparation kit, and sequenced on an Illumina NextSeq500 instrument as previously described (3). Library preparation and sequencing were performed at the Research Resources Center, University of Illinois at Chicago. *De novo* assembly was performed with the SPAdes assembler (4). Assembly coverage statistics were computed by mapping raw reads to the assembled genome using Bowtie 2 (5); low-coverage contigs were removed. Genome islands were identified by Island Viewer (6), and phylogenetic affiliation of strain B1 was assessed via the One Codex (https://www.onecodex.com/) database.

Approximately 87.5% of genomic reads from strain B1 were taxonomically affiliated with Pseudomonas sp. strain GM33, but 16S rRNA PCR amplicon sequencing suggested strain B1 to be closer to Pseudomonas reinekei strain LBUM376. The genome sequence, with an *N*_50_ value of 300,412 and an *L*_50_ of 9, spanning over 48 contigs at 500× coverage, was then annotated by RAST (7). The genome size of strain B1 was 6,706,934 bases (https://figshare.com/s/1392dba833810b30ac0d), with 6,059 coding sequences and a G+C content of 60.3%. RAST identified 545 subsystems from strain B1 (https://figshare.com/s/9fa5cc9c2b185b28a614), with a suite of genes (count in parentheses) for membrane transport (229), stress response (222), metabolism of aromatic compounds (175), and motility and chemotaxis (124). Moreover, several gene homologues previously implicated in heavy metal/radionuclide resistance were also identified, including the cobalt-zinc-cadmium efflux system, membrane transporters, metalloproteases, and antimicrobial extrusion proteins; these genomic features likely facilitate B1’s survival within the contaminated EFPC sediments.

Interesting evolutionary traits, such as the presence of several genomic islands (GEIs), were also identified in strain B1. GEIs provide genome plasticity and beneficial traits to the host for survival in stressful environments (8). When evaluated against the genome of the metabolically versatile Pseudomonas putida KT2440, strain B1 was found to be interspersed with many GEIs (https://figshare.com/s/7ff712b74791052c637b), with several genes for biodegradative and metal homeostatis functions. These findings suggest that GEIs were likely horizontally acquired by strain B1 for survival in the contaminated EFPC habitat.

Overall, we show that sediment-borne pseudomonads, such as strain B1, harbor a plethora of ecologically relevant genomic features, an observation that is in line with those reported in previous studies (9, 10). Such studies provide genomic insights into the underpinnings of bacterially mediated bioremediative functions and future recommendations for better stewardship of heavy-metal-contaminated ecosystems.

### Accession number(s).

The whole-genome shotgun project of the Pseudomonas isolate reported in this study has been deposited at DDBJ/ENA/GenBank under the accession number PYUK00000000.

## References

[1] Brooks SC, Southworth GR 2011 History of mercury use and environmental contamination at the Oak Ridge Y-12 plant. Environ Pollut 159:219–228. doi:10.1016/j.envpol.2010.09.009. 20889247

[2] Hughes J 2008 Oak Ridge Reservation annual site environmental report for 2008. DOE/ORO/2296 Oak Ridge National Laboratory, Oak Ridge, TN.

[3] Chauhan A, Pathak A, Jaswal R, Edwards B III, Chappell D, Ball C, Garcia-Sillas R, Stothard P, Seaman J 2018 Physiological and comparative genomic analysis of *Arthrobacter* sp. SRS-W-1-2016 provides insights on niche adaptation for survival in uraniferous soils. Genes 9:31. doi:10.3390/genes9010031. PMC5793183 29324691

[4] Bankevich A, Nurk S, Antipov D, Gurevich AA, Dvorkin M, Kulikov AS, Lesin VM, Nikolenko SI, Pham S, Prjibelski AD, Pyshkin AV, Sirotkin AV, Vyahhi N, Tesler G, Alekseyev MA, Pevzner PA 2012 SPAdes: a new genome assembly algorithm and its applications to single-cell sequencing. J Computational Biology 19:455–477. doi:10.1089/cmb.2012.0021. PMC3342519 22506599

[5] Langmead B, Salzberg SL 2012 Fast gapped-read alignment with Bowtie 2. Nat Methods 9:357–359. doi:10.1038/nmeth.1923. 22388286 PMC3322381

[6] Bertelli C, Laird MR, Williams KP; Simon Fraser University Research Computing Group, Lau BY, Hoad G, Winsor GL, Brinkman FSL 2017 IslandViewer 4: expanded prediction of genomic islands for larger-scale datasets. Nucleic Acids Res 45:W30–W35. doi:10.1093/nar/gkx343. 28472413 PMC5570257

[7] Aziz R, Bartels D, Best A, DeJongh M, Disz T, Edwards R, Formsma K, Gerdes S, Glass E, Kubal M, Meyer F, Olsen G, Olson R, Osterman A, Overbeek R, McNeil L, Paarmann D, Paczian T, Parrello B, Pusch G, Reich C, Stevens R, Vassieva O, Vonstein V, Wilke A, Zagnitko O 2008 The RAST Server: rapid annotations using subsystems technology. BMC Genomics 9:75. doi:10.1186/1471-2164-9-75. 18261238 PMC2265698

[8] Juhas M, van der Meer JR, Gaillard M, Harding RM, Hood DW, Crook DW 2009 Genomic islands: tools of bacterial horizontal gene transfer and evolution. FEMS Microbiol Rev 33:376–393. doi:10.1111/j.1574-6976.2008.00136.x. 19178566 PMC2704930

[9] Silby MW, Winstanley C, Godfrey SA, Levy SB, Jackson RW 2011 *Pseudomonas* genomes: diverse and adaptable. FEMS Microbiol Rev 35:652–680. doi:10.1111/j.1574-6976.2011.00269.x. 21361996

[10] Cánovas D, Cases I, de Lorenzo V 2003 Heavy metal tolerance and metal homeostasis in *Pseudomonas putida* as revealed by complete genome analysis. Environ Microbiol 5:1242–1256. doi:10.1111/j.1462-2920.2003.00463.x. 14641571

